# A Retrospective Analysis of Indoor CO_2_ Measurements Obtained with a Mobile Robot during the COVID-19 Pandemic

**DOI:** 10.3390/s24103102

**Published:** 2024-05-13

**Authors:** Jordi Palacín, Elena Rubies, Eduard Clotet

**Affiliations:** Automation and Robotics Laboratory (ARL), Universitat de Lleida, 25001 Lleida, Spaineduard.clotet@udl.cat (E.C.)

**Keywords:** CO_2_ sensor, mobile robot, air quality, COVID-19, airborne outbreak

## Abstract

This work presents a retrospective analysis of indoor CO_2_ measurements obtained with a mobile robot in an educational building after the COVID-19 lockdown (May 2021), at a time when public activities resumed with mandatory local pandemic restrictions. The robot-based CO_2_ measurement system was assessed as an alternative to the deployment of a net of sensors in a building in the pandemic period, in which there was a global stock outage of CO_2_ sensors. The analysis of the obtained measurements confirms that a mobile system can be used to obtain interpretable information on the CO_2_ levels inside the rooms of a building during a pandemic outbreak.

## 1. Introduction

The concept of indoor air quality is generally associated with the health and comfort of a building’s occupants [1]. Carbon dioxide (or CO_2_) is a chemical compound, produced as a byproduct of the breathing process, that must be analyzed when assessing air quality [2] due to its negative effects on human health, particularly when present in high concentrations [3,4,5,6,7,8]. On average, humans produce around 100 times the original quantity of inhaled CO_2_ [3], a high exchange ratio that can contribute to a noticeable rise in CO_2_ concentrations, especially in crowded or poorly ventilated indoor spaces. This highlights the importance of keeping an adequate ventilation regime in shared indoor spaces, which is not a trivial problem. In this context, Ding et al. [8] reviewed the classroom ventilation regimes of several educational buildings, concluding that unsupervised ventilation strategies are not sufficient to guarantee a healthy environment for students.

The concentration of CO_2_ measured inside a room can be used as a biomarker to estimate if the air inside the room is adequately renewed to prevent the build-up of undesired gases [9,10]. Di Gilio et al. [11] proposed the implementation of specific threshold levels to evaluate the risk of inhaling the air exhaled by others in a poorly ventilated room, which should be used as a trigger mechanism to set corrective actions.

In relation to the global pandemic of coronavirus disease 2019 (COVID-19) that was caused by severe acute respiratory syndrome coronavirus 2 (SARS-CoV-2), the scientific literature [12,13,14] highlighted that the most common infection pathway was the inhalation of contagious airborne particles in the form of small droplets or aerosols [15], which are ejected from the mouth and nose of the infected person via the respiratory system while breathing, talking, or sneezing [16]. Zhang et al. [12] analyzed the potential infection risks of people who were exposed to the SARS-CoV-2 virus in public places and non-healthcare environments, concluding that inhalation was the predominant route of exposure, emphasizing the importance of protecting individuals from the airborne transmission of infectious diseases.

In the rise of the COVID-19 pandemic, the World Health Organization (WHO) [17] highlighted that “the risk of getting COVID-19 is higher in crowded and inadequately ventilated spaces where infected people spend long periods of time together in close proximity”. The WHO suggested the application of some preventive measures, such as an increase in natural indoor ventilation by opening doors and windows, which were implemented worldwide [4]. However, the analysis of the ventilation regimes performed by Fantozzi et al. [4] concluded that, even in naturally ventilated buildings, it is generally not possible to estimate the amount of fresh air entering shared indoor spaces without the use of dedicated ambient sensors measuring the concentration of indoor air pollutant gases such as CO_2_.

After vaccination [18,19], indoor air renovation is considered one of the most important factors for reducing the probability of massive airborne pathogen contagion when individuals spend long periods of time indoors [20,21,22,23]. The CO_2_ concentration measured inside a room can also be used as a biomarker of the airborne pathogen transmission [24,25,26,27,28]. At the time that the CO_2_ measurements reported in this paper were performed, the strategy applied to reduce the risk of airborne transmission of the SARS-CoV-2 virus was based on the risk classification scheme proposed by Di Gilio et al. [11], which was derived from the analytical expressions proposed by Peng et al. [27]. One of the assumptions of Peng et al. [27] was that no virus remains infectious in the air after a few hours of inactivity [29], so there is a background or bias CO_2_ concentration with no risk of pathogen transmission, as only the new excess of exhaled CO_2_ generated during the activity contributes effectively to increasing the risk of infection. More information on specific studies modeling the airborne transmission of COVID-19 in built environments can be found in Vouriot et al. [30] and in the review presented by Rayegan et al. [31].

### 1.1. New Contribution

This paper presents a retrospective analysis of the indoor CO_2_ concentrations obtained with a mobile robot during the COVID-19 pandemic. The measurements were carried out in May 2021 after lockdown, at a time when public activities resumed and there was a global stock outage of CO_2_ sensors [32] because of the COVID-19 supply chain crisis [33]. The starting hypothesis of this work was that an autonomous mobile robot with an embedded CO_2_ sensor can be used as an alternative to the deployment of a net of sensors to measure the CO_2_ levels across different rooms of a building.

According to Wang et al. [34], who reviewed 280 scientific works describing robot technologies during the COVID-19 pandemic, there were no reports on robot-based CO_2_ measurement systems, so this retrospective analysis aims to revise and share the CO_2_ measurements obtained with a mobile robot during the COVID-19 pandemic. The main specificities of the robot-based CO_2_ measurement system are its human size, its interactive capability, its omnidirectional mobility, and its ability to navigate between floors of a multi-story building.

### 1.2. Structure of the Paper

This paper is structured as follows: Section 2 describes the background of CO_2_ sensors and CO_2_ measurements with mobile platforms. Section 3 presents the materials and methods used in this work. Section 4 describes the experiments conducted and the CO_2_ concentrations obtained in the experiments. Section 5 presents a retrospective analysis of the results. Section 6 discusses the results obtained, provides the conclusions of the paper, and presents possible future developments.

## 2. Background

### 2.1. CO_2_ Sensors

There are a wide variety of sensing technologies that can be used to measure indoor CO_2_ concentrations [35]. A popular option is based on the use of non-dispersive infrared (NDIR) laser reflection, which provides a direct measurement of the CO_2_ concentration that is highly uncorrelated with other ambient parameters such as humidity [35]. The development of the Internet of Things (IoT) [36,37,38] has motivated a special tendency towards the deployment of de-centralized sensors for indoor air quality monitoring [39], mainly focused on the use of low-cost [40], battery-powered sensing devices [41]. As an example of an IoT application, Marques et al. [39] proposed a custom air quality sensor based on CO_2_ analysis that could be deployed in large buildings. The measurements gathered by the device can be accessed through a web or smartphone application. In a similar direction, Vanhaeverbeke et al. [42] proposed a real-time IoT software architecture to calculate and visualize the estimated risk of COVID-19 aerosol transmission in office buildings, using colored lights placed in each room. Their platform was able to process large volumes of data gathered by hundreds of sensors in real-time.

The deployment of a net of stationary CO_2_ sensors in an existing building has some drawbacks [43] that should not be underestimated, such as cost, scalability, deployment time, and maintenance. For example, the medium-sized educational building in which the experimental part of this paper was performed would require the deployment of a net composed of 95 stationary CO_2_ sensing devices to monitor all its shared spaces. In this context, the use of mobile robots carrying high-quality CO_2_ sensors can be an efficient alternative to monitor air quality, especially when taking into consideration the time and cost associated with the deployment of the measurement system.

### 2.2. CO_2_ Measurement with Mobile Platforms

The measurement of CO_2_ with a mobile platform has been addressed by several authors [44,45,46,47,48]. Jin et al. [44] proposed the development of an automated mobile sensing platform for high-granularity indoor environmental quality monitoring. Quintana et al. [45] proposed using several sensors embedded in a small, low-cost, remotely controlled mobile robot for workplace occupant comfort monitoring. Rahmaniar et al. [46] proposed a hybrid approach, in which a mobile robot was used to carry an air quality sensor connected wirelessly to a base station. Yang et al. [47] proposed the use of a mobile robot to detect and locate a CO_2_ contaminant source, obtaining better localization accuracy than when using a net with nine stationary sensors. Curea et al. [48] proposed a semi-autonomous omnidirectional mobile robot to monitor a wide range of ambient parameters, including CO_2_ and other harmful industrial gases.

In general, the use of a mobile platform for indoor CO_2_ monitoring has the disadvantage that only one location can be analyzed at a time. However, the use of an autonomous mobile robot with an embedded CO_2_ sensor also has some advantages: the granularity of the measurements is higher than when using stationary sensors; the calibration and maintenance is simpler; the onboard computational power of the mobile robot can be used to estimate other parameters such as the occupancy of the room and type of activity; the patrolling route can be optimized depending on the planned room occupancy; and CO_2_ monitoring can be performed as a secondary task.

The advances of robotic technologies during the COVID-19 pandemic have been analyzed by several authors [34,49]. Specifically, Wang et al. [34] reviewed 280 scientific works and discussed 146 of them in detail to identify representative technologies and their readiness level. There were no reports on robot-based CO2 measurement systems used as a preventive tool to fight the transmission of the COVID-19 pandemic. As stated before, this gap inspired the revision of the measurements obtained with a mobile robot embedding a CO_2_ sensor during the COVID-19 pandemic.

## 3. Materials and Methods

This section describes the materials used in this work: the mobile robot used to carry a CO_2_ sensor and perform the measurements, the selected CO_2_ sensor, and the methods used for map creation, path-planning, self-localization, path-tracking, and robot-to-building integration.

### 3.1. Mobile Robot

**Figure 1:**
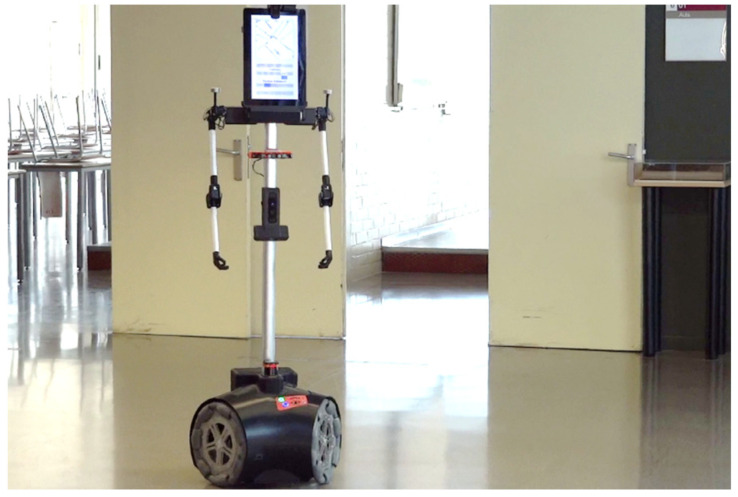
Mobile robot performing a routine inspection.

Figure 1 shows the mobile robot used in this work, which was developed by the Automation and Robotics Laboratory of the University of Lleida, Spain [50]. This omnidirectional humanoid-like robot (1.76 m, 30 kg) was initially designed to provide assistive services [51] and interact with people [52]. The robot embeds a Hokuyo UTM-30LX high-performance 2D LIDAR (Hokuyo, Osaka, Japan), which is based on an internal rotating laser-ranging sensor, to conduct a planar 2D exploration (or scan) of the area surrounding the sensor. This LIDAR has a radial distance range of up to 40 m, with a precision between 30 and 50 mm, covers a plane of 270° around the sensor, and provides 1081 points per scan, at a rate of 40 scans/s. The mobile robot processes the information gathered by this 2D LIDAR for simultaneous localization and mapping (SLAM) [53], obstacle avoidance [54,55], and autonomous path-planning and path-tracking.

The robot (Figure 1) has a tactile screen for human interaction [52], several onboard cameras, redundant distance sensors, passive infrared detectors (PIRs) for detecting occupancy, and two articulated arms with figurative fixed hands. In this work, the mobile robot embeds a CO_2_ sensor at a height of 1.3 m above floor level. Preliminary tests showed that placing the sensor at different heights in the structure of the robot did not influence the registered CO_2_ levels.

### 3.2. CO_2_ Sensor Embedded in the Mobile Robot

There are different sensing technologies that can be used to measure CO_2_ concentrations under indoor conditions [35]. The sensor embedded in the mobile robot is based on non-dispersive infrared (NDIR) technology. The non-dispersive infrared (NDIR) CO_2_ sensor is the S8-LP (Senseair, Stationsgatan 12, 824 71 Delsbo, Sweden), which has an accuracy of ±40 ppm in the measurement ranges from 400 to 2000 ppm and ±3% of reading in ranges from 0 to 10,000 ppm. This sensor requires a warm-up time of at least 30 s after a cold start and measures CO_2_ concentration, temperature, and humidity once every second. This sampling time was considered adequate for mobile CO_2_ monitoring.

### 3.3. Method for 2D Map Creation

The deployment of a mobile robot in a building requires a map for planning the exploratory missions, calculating the optimal path to fulfill a mission, self-estimating its relative position and orientation, and tracking the planned path.

**Figure 2:**
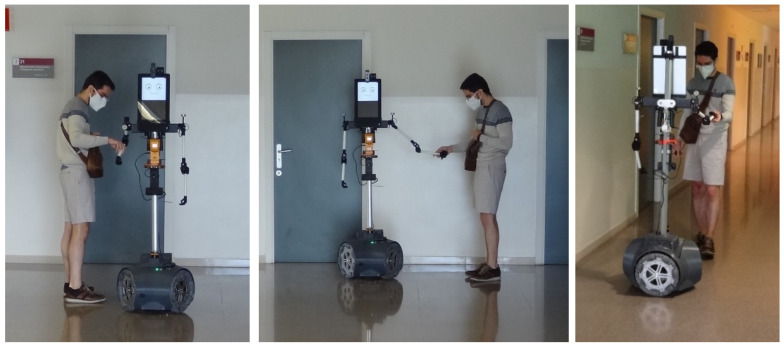
Images of one of the authors of this work manually guiding the robot while performing the supervised exploration of a building during the COVID-19 pandemic.

The map used by the robot is a 2D point cloud map of the building. In this case, the 2D point cloud map was obtained in a supervised exploration of the building performed before starting the measurements, during a public holiday with no people in the building. The creation of a map requires the following steps: a complete exploration of the building while recording the scans gathered by the 2D LIDAR and the odometry data, automatic registration and merging of the 2D scans in a single 2D map, manual refining of the map, automatic creation of the navigation tree of the map, and manual definition of the reference waypoints used to define the exploratory missions. During this supervised exploration of the building, the robot was manually guided (see Figure 2) [51] to ensure that all educational classrooms were explored. This map only needs to be created once and can be used by all mobile robots operating in the building [56].

**Figure 3:**
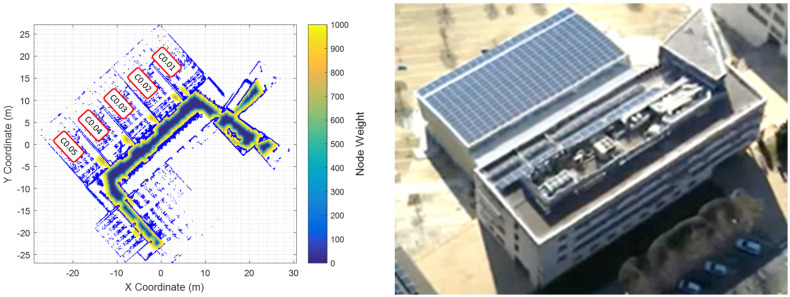
Two views of the experimentation building: (**left**) point cloud map of its first floor including classroom numbers and a color scale identifying the navigable areas (colored nodes), and (**right**) 3D photometric reconstruction of the exterior of the building.

The final 2D point cloud map is created by joining the 2D scans obtained during the exploration by using a variant of the Iterative Closest Point (ICP) algorithm [54,57]. The result of this matching process is the determination of a common reference coordinate system for all the scans, allowing them to be unified in a single 2D map. Figure 3 shows the map and the exterior of the educational building explored in this work, which is the Polytechnic School of the Universitat de Lleida, Spain. At this point, the consistency of the map created from a 2D LIDAR depends on the physical characteristics of the walls and furniture elements being scanned [58]. In general, the raw map of a facility requires some manual refining to create enclosed areas with the use of virtual walls. This feature is essential, since some obstacles, such as glass panels, hollow handrails, or stairwells, may not be detectable with a 2D LIDAR. Virtual walls created during the manual refinement of the map can also serve to restrict the navigable areas that are accessed by the robot.

In this work, the navigable areas are defined by a grid with nodes measuring 0.120 m × 0.120 m each, which is created using the Breadth-First Search (BFS) algorithm [59]. This algorithm initiates at the robot’s initial coordinates at the start of the exploration and iteratively expands to neighboring nodes until all accessible areas are mapped, avoiding contact with the mapped wall points. Then, each node of the grid is weighted according to its distance to the nearest obstacle or wall. The weights of the nodes are represented in Figure 3 using a color scale: the blue color represents the safest navigable area, while the yellow color represents areas that, although navigable, are much closer to obstacles.

The navigable areas are used to create the navigation tree of the map, which represents the list of interconnections between all its nodes. The granularity of the grid and the connectivity of the nodes define the computational challenge that represents the use of deterministic search algorithms [60] to plan the trajectory of the robot. However, recent works have demonstrated that similar navigation results can be obtained when using a navigation tree that is not based on a discrete grid definition [61].

Finally, the map must be edited to label the nodes depicting relevant destinations (points of interest) such as charging stations, classrooms, elevators, etc. The use of labeled nodes simplifies the process of defining routes and managing the connections between important locations, especially in the case of elevators, which are used as transition points between different floors.

### 3.4. Method for Path-Planning

The path-planning algorithm is responsible for computing a sorted list of nodes to be visited in order to reach the destination defined in a mission [62]. The path-planning method used in this work to explore the navigation tree of the building is the A* (A-star) algorithm [60,62,63]. Starting from a known waypoint (or node position) on the map, the A* algorithm determines which sequence of nodes in the navigation tree are to be visited to complete an exploratory mission. This sequence includes the possibility of accessing elevators to navigate between the floors of the building during a mission [64].

**Figure 4:**
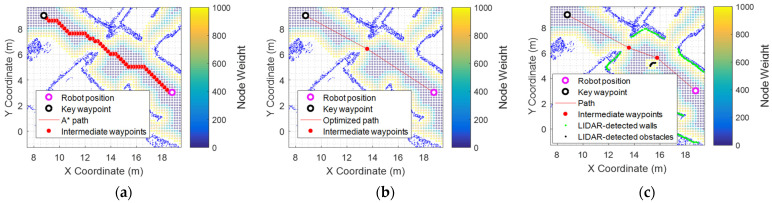
Path-planning example. The blue dots represent the point cloud of the map, while the green dots represent the current 2D scan points that match with the map: (**a**) the discrete trajectory found with the A* algorithm to navigate to a key waypoint using the navigation tree with a grid of 0.12 m × 0.12 m; (**b**) the optimized path avoiding unnecessary intermediate maneuvers; and (**c**) the recalculated path to avoid an obstacle (black points) detected in the planned path.

Figure 4 depicts an example of the planned path from the current robot position (violet ring) to a destination defined by a key waypoint (black ring). Figure 4 shows small colored circles detailing the localization of the nodes of the navigation tree in the area. The direct result of the A* algorithm (Figure 4a) initially forces the robot to move in node-sized steps to visit one of its eight neighbors. This result is then smoothed to reduce the total number of nodes to be visited (Figure 4b) [65] while ensuring the safety of the robot [63].

The robot has been designed to operate in dynamic environments with ongoing human activity, so it is expected that new static and dynamic obstacles will be encountered during exploratory missions. As an application example, Figure 4c represents the detection of an obstacle (black dots) in the planned path, which triggers the computation of a new trajectory to avoid the obstacle. In this case, the onboard computational power of the robot enables real-time re-evaluation of the path by executing the A* algorithm on the spot to automatically update the planned path upon detecting an obstacle.

### 3.5. Methods for Self-Localization and Path-Tracking

The method employed for robot self-localization relies on matching the current 2D scan provided by its 2D LIDAR sensor with the 2D map of the building floor by using the ICP algorithm [54,57]. This matching provides an estimate of the relative location and orientation of the robot on the 2D map. The obstacle detection procedure implemented in the robot is performed after the ICP matching [54,66]. The points of the scan retrieved by the LIDAR sensor which have not been matched with other points of the map are automatically identified as obstacles to be avoided.

The path-tracking procedure implemented in the robot is described in [67]. This procedure is based on sampling the planned path to define a sequence of intermediate trajectory waypoints to be visited by the omnidirectional robot.

### 3.6. Method for Robot-to-Building Integration

At the time when this work was performed, it was not possible to interact or send information to the mechanical ventilation system of the building. As an alternative, the robot was configured to send warning messages to a remote operator when one of the monitored parameters exceeded a predefined threshold. Therefore, if necessary, the alerted remote operator would re-adjust the heating, ventilation, and air conditioning (HVAC) system of the building to implement corrective measures. The technical challenges and benefits of an interactive relationship between a mobile sensing platform and the existing demand-driven HVAC [68] of a building will be explored in future works.

## 4. Results

### 4.1. Exploratory Missions

Table 1 summarizes the exploratory missions scheduled to register the CO_2_ levels in five rooms of an educational building. Classrooms C0.01, C0.02, C0.03, C0.04, and C0.05 (defined in Figure 3) have a similar size, layout, furniture distribution, entrance door, and windows. The classrooms are designed to carry out quiet lectures, an educational activity involving no physical activity, and in which only the lecturer is usually speaking or moving in front of a blackboard. For the purpose of mobile robot exploration, each classroom is identified by a node-label or waypoint, which is physically defined not too far into the classroom to minimize possible lecture disruptions while performing CO_2_ measurements.

Table 1 defines two different missions. The first mission M0, scheduled at 8:00 h, was proposed to validate the correct operation of the robot before starting the educational activities in the building and to record reference or background CO_2_ levels. The following missions, from M1 to M4, were scheduled throughout the morning to register the CO_2_ level inside the classrooms in accordance with the planned occupancy. In all these missions, the robot started from a charging station, tracked the planned trajectory, stopped after reaching the coordinates of the designated point of interest inside the different classrooms, waited for 5 min in order to obtain a stable CO_2_ measurement, and returned to the charging station. During the exploratory missions, the mobile robot was instructed to move at a medium–low translational velocity (0.2 m/s) and configured in silent mode with no sound generation.

### 4.2. CO_2_ Measurement Results

This section presents the CO_2_ levels obtained by the mobile platform while performing the exploratory missions M0 to M4. The measurements were carried out in May of 2021, after a local COVID-19 lockdown, when activities were gradually resumed with the compulsory pandemic restrictions applied [22,69]. Specifically, these were as follows: windows and doors open, 10 min break after 50 min of activity, students seated at least one meter away from each other, and compulsory use of face masks. In such pandemic conditions, all the doors of the classrooms that the robot encountered on its exploration path were open, and using a mobile robot to enter classrooms to monitor the CO_2_ level was perceived as safer than if this operation were performed by a human operator.

**Figure 5:**
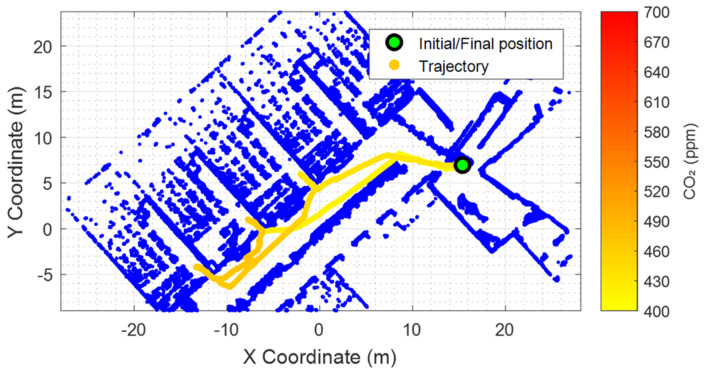
Georeferenced representation of the CO_2_ concentration evolution recorded by the mobile robot during the M0 exploratory mission.

**Figure 6:**
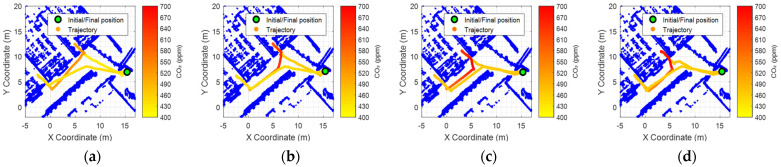
Georeferenced representations of the CO_2_ concentration levels registered by the mobile robot during the exploratory missions (**a**) M1, (**b**) M2, (**c**) M3, and (**d**) M4.

Figure 5 shows the georeferenced evolution of the CO_2_ levels registered during the M0 mission. The CO_2_ levels registered ranged from 404 ppm in the hallway to 468 ppm inside C0.05 classroom. Similarly, Figure 6 shows the georeferenced evolution of the CO_2_ levels registered during the M1, M2, M3, and M4 missions. Figure 6 illustrates the presence of inertia in the measurements obtained from the CO_2_ sensor, but the 5 min waiting period inside the classrooms was deemed sufficient for obtaining valid measurements.

**Figure 7:**
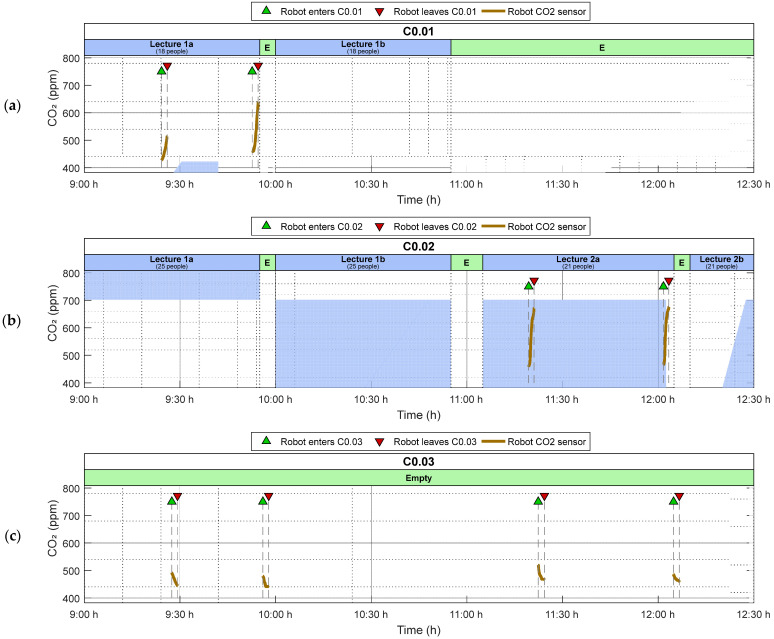
Occupancy and CO_2_ levels registered by the mobile measurement system in (**a**) C0.01 classroom; (**b**) C0.02 classroom; and (**c**) C0.03 classroom.

The outcomes of M1, M2, M3, and M4 missions are also presented in Figure 7, employing a time scale evolution to evince that the mobile robot provides sampled information on the CO_2_ levels inside the explored classrooms. Figure 7a shows the CO_2_ levels obtained inside C0.01 classroom during two exploratory missions (M1 and M2) and the number of people attending the lecture. During the 5 min wait, the concentration increases and stabilizes, although the time scale of the figure makes it difficult to observe this stabilization. Figure 7a shows that M2 mission registers a higher CO_2_ level inside the room, revealing the cumulated effect of the CO_2_ exhaled during the lecture (despite having the windows and door open). Similarly, Figure 7b shows the CO_2_ levels obtained inside C0.02 classroom during two exploratory missions (M3 and M4) and the number of people attending the lecture. In these two measurements, the CO_2_ concentration stabilized at nearly the same level, probably due to an equilibrium between the exhaled CO_2_ during the lecture and the inflow of fresh air into the room. Finally, Figure 7c shows the CO_2_ levels obtained inside C0.03 classroom during the four M1 to M4 exploratory missions, which was empty throughout the day. The sensor needs some time to show the change in CO_2_ concentration inside the classroom. However, after the 5 min wait, the registered CO_2_ level is practically the same in all the missions.

### 4.3. Evaluation of the Risk of COVID-19 Infection

At the time when the exploratory missions were conducted (May 2021), the evaluation of the risk of COVID-19 infection was based on the safe threshold CO_2_ levels proposed by Di Gilio et al. [11] to reduce the risk of SARS-CoV-2 airborne transmission (shown in Table 2). These threshold levels can be adapted depending on the pathogen transmitted [11].

Table 3 shows the classroom monitored, the mission time, the expected classroom occupancy, the cumulated occupancy time, the maximum CO_2_ levels registered by the robot, and an estimation of the risk of COVID-19 infection according to the safe threshold levels applied. The maximum CO_2_ levels registered in all the monitored classrooms were below 700 ppm, so the risk of SARS-CoV-2 airborne transmission in the occupied classrooms was always evaluated as “Low Risk”.

## 5. Retrospective Analysis

The development of this work has provided an opportunity to revise the CO_2_ levels obtained in May of 2021. The objective of this section is to provide an updated evaluation of the risk of COVID-19 infection. This analysis is inspired by the work by Vanhaeverbeke et al. [42], who in 2023 proposed a real-time estimation of the risk of airborne transmission of SARS-CoV-2 in an office building. Their risk evaluation was conducted using the model proposed by Bazant et al. [70] to dynamically estimate a safe CO_2_ excess limit (relative to the background CO_2_ level) to mitigate SARS-CoV-2 aerosol transmission, accounting for factors such as occupancy and cumulated exposure time. The assumption is that no virus remains infectious in the air after a few hours of inactivity [29], so there is a background or bias CO_2_ concentration with no risk of pathogen transmission (registered during M0 mission), as only the new excess of exhaled CO_2_ during an activity contributes to effectively increase the risk of infection. The safe CO_2_ concentration excess estimated by the model is not a fixed value, decreasing exponentially as the exposure time increases [70].

The model proposed by Bazant et al. [70] has the advantage of providing a safe CO_2_ excess value that can easily be compared with the CO_2_ concentration registered during an indoor activity. This model requires a large list of input parameters that must be accurately determined to obtain a reliable estimation. The input parameters of the model are as follows: the size of the room, the characteristics of the ventilation and air renewal in the room, the type of activity (quiet or dynamic), and other specific information regarding the people involved in the activity taking place in the room, namely, the number of people, age range, use of masks, expected percentage of people infected with COVID-19, and expected percentage of people with COVID-19 immunity. Bazant et al. [71] provide useful case examples and a spreadsheet to guide and simplify the application of the model. Nevertheless, this model can be replaced with any other practical indicator that has been adapted to the current airborne pandemic [72].

The maximum CO_2_ level obtained during the M0 mission (468 ppm, see Table 3) was used to set the background CO_2_ level for the classrooms to compute the exhaled CO_2_ excess generated inside the classrooms. Table 4 details the maximum CO_2_ excess registered inside the classrooms (relative to the reference background CO_2_ level of 468 ppm). Table 4 also details the type of activity performed in the classrooms, along with the use of face masks by classroom users, which was 100% due to local regulations that made wearing them compulsory in that period (May 2021) [22]. The expected number of infected people (presumably asymptomatic) in the classrooms was obtained from governmental data sources offering public average regional information [73] and was rounded to the upper integer: one person for the occupancy expected in the classrooms. Similarly, the percentage of immune people in the classrooms was also obtained from governmental data sources [74,75]. In this case, the availability of official data sources offering regional information of the evolution of the COVID-19 pandemic [73,74,75] avoided the task of collecting individual information directly from the people present in the classroom activities.

Table 4 details the value of the safe CO_2_ excess limit estimated with the model proposed by Bazant et al. [70]. In the case of C0.01 classroom, this value is higher than 1600 ppm for an occupancy time of up to 1 h, dropping to 1325 ppm after 2 h. In the case of C0.02 classroom, this value is 891 ppm for an occupancy time of 3 h. Comparatively, these safe values are significantly higher than the absolute threshold levels defined by Di Gilio et al. [11], since the dynamic model [70] also includes the filtering effect provided by the use of face masks, which significantly increases the safe CO_2_ threshold level during an indoor activity [76,77].

Finally, Table 4 provides an estimation of the risk of SARS-CoV-2 aerosol transmission based on the proposal of Vanhaeverbeke et al. [42]: dividing the measured CO_2_ excess by the value of the safe CO_2_ excess provided by the model [70] and clipping the results in a range from 0 (0% risk) to 1 (100% risk). Therefore, a risk of 100% means that the safe CO_2_ excess of the indoor activity has been surpassed, so that a healthy, non-immune occupant of the room is at risk of being infected by airborne transmission of SARS-CoV-2. The risks that were finally assessed were 0% for an occupancy time up to 1 h, rising to 15% after 2 h and to 25% after 3 h. These risk values confirm the adequacy of the measures deployed to reduce the risk of airborne transmission of the SARS-CoV-2 virus [22].

## 6. Discussion and Conclusions

This paper presents the work carried out with an autonomous robot-based measurement system embedding a CO_2_ sensor during the COVID-19 pandemic. This work was developed at a time when there was a global stock outage of CO_2_ sensors [32]. The hypothesis was that a mobile measurement system can be used as an alternative to the deployment of a net of sensors to measure the CO_2_ levels of different rooms in a building.

Table 5 compares the mobile measurement system deployed in this work with the referenced robots embedding a CO_2_ sensor [44,45,46,47,48]. The main differential elements of the proposed robot are its size (1.76 m, 30 kg), its human interaction capability [52], and its omnidirectional mobility [50]. The robot also has the ability to navigate between the floors of a multi-story building [61].

A series of experiments were conducted in an educational building to measure the CO_2_ levels in different classrooms with the robot. These experiments were performed in May of 2021 after a local COVID-19 lockdown, when activities resumed following the mandatory pandemic restrictions established by the government [22,69]. During the experiments, all the doors of the classrooms that the robot encountered on its exploration path were open, and the use of a mobile robot to monitor the CO_2_ levels was perceived as safer compared to human operators performing the same monitoring task.

The CO_2_ levels obtained during the explorations were evaluated in real-time according to the risk classification scheme proposed by Di Gilio [11] to reduce the risk of SARS-CoV-2 airborne transmission. All monitored classrooms received the classification of “Low Risk” of SARS-CoV-2 airborne transmission (see Table 3).

This work also presents a retrospective analysis of the CO_2_ levels registered. This new analysis is inspired by the work carried out in 2023 by Vanhaeverbeke et al. [42], which evaluated the risk of COVID-19 airborne transmission from real-time CO_2_ measurements based on the mathematical model proposed by Bazant et al. [70,71]. This model provides an upper bound of the CO_2_ excess, tailored to prevent aerosol transmission of SARS-CoV-2 in indoor environments, depending on the occupancy and time cumulated in a room. The application of this model showed a maximum risk of SARS-CoV-2 airborne transmission in the classrooms of 25% (see Table 4), a value that confirms the adequacy of the pandemic restrictions applied in the building.

The conclusion of this work is that a mobile measurement system can be used to obtain interpretable information on the CO_2_ levels inside the rooms of a building. The development of mobile CO_2_ monitoring capabilities is aligned with the advice of the World Health Organization (WHO) [78], highlighting that “future pandemics are inevitable”. The use of the robot to estimate the size and occupancy of a room, as well as the use of face masks, will be studied in future works. The measurement system will be adapted to improve the control and sustainability of the management of the HVAC [68,79]. Finally, another promising improvement is the integration of a CO_2_ sensor in a compact eNose, trained to operate fully as an early non-invasive detector of airborne transmitted pandemic outbreaks [80,81].

## Figures and Tables

**Table 1:** Exploratory missions scheduled.

Mission	Scheduled	Description	Rooms to Explore
M0	08:00 h	Robot test and obtain reference background CO_2_ levels	C0.03; C0.04; C0.05
M1	09:20 h	Normal classroom exploration	C0.01; C0.03
M2	09:50 h	Normal classroom exploration	C0.01; C0.03
M3	11:18 h	Normal classroom exploration	C0.02; C0.03
M4	12:00 h	Normal classroom exploration	C0.02; C0.03

**Table 2:** Risk classification scheme proposed by Di Gilio [11] to reduce the risk of SARS-CoV-2 airborne transmission based on CO_2_ concentration monitoring.

	Low Risk	Moderate Risk	High Risk	Very High Risk
CO_2_ concentration	<700 ppm	>700 ppm	>800 ppm	>1000 ppm
		<800 ppm	<1000 ppm	

**Table 3:** Estimation of the risk of COVID-19 infection based on the safe CO_2_ threshold levels proposed by Di Gilio et al. [11] (shown in Table 2).

Mission: Classroom	Exploration Time	Occupancy (People)	Occupancy Time	Maximum CO_2_ Level Registered	Risk Inhaling the Air Exhaled by Other Occupants [11]
M0: C0.03	08:02 h	0	0 h	448 ppm (Figure 5)	-
M0: C0.04	08:08 h	0	0 h	453 ppm (Figure 5)	-
M0: C0.05	08:14 h	0	0 h	468 ppm (Figure 5)	-
M1: C0.01	09:22 h	18	00:22 h	526 ppm (Figure 6a)	Low Risk
M2: C0.01	09:50 h	18	00:50 h	650 ppm (Figure 6b)	Low Risk
M3: C0.02	11:18 h	21	02:18 h	670 ppm (Figure 6c)	Low Risk
M4: C0.02	12:02 h	21	03:02 h	689 ppm (Figure 6d)	Low Risk
M1: C0.03	09:28 h	0	0 h	445 ppm (Figure 6a)	-
M2: C0.03	09:56 h	0	0 h	455 ppm (Figure 6b)	-
M3: C0.03	11:24 h	0	0 h	473 ppm (Figure 6c)	-
M4: C0.03	12:08 h	0	0 h	466 ppm (Figure 6d)	-

**Table 4:** Estimation of the risk of SARS-CoV-2 aerosol transmission based on the CO_2_ excess registered and the model of the safe CO_2_ excess proposed by Bazant et al. [70].

CO_2_ Measurements		Bazant et al. [70] Model					Risk of SARS-CoV-2 Aerosol Transmission [42]
		Inputs				Output	
Mission: Classroom	CO_2_ Excess Registered	Type of Activity	Use of Face Masks	People Infected	People Immune	Safe CO_2_ Excess	
M1: C0.01	58 ppm	Quiet	100% ^A^	1 ^B^	10% ^B^	>1600 ppm	0%
M2: C0.01	128 ppm	Quiet	100% ^A^	1 ^B^	10% ^B^	>1600 ppm	0%
M3: C0.02	202 ppm	Quiet	100% ^A^	1 ^B^	10% ^B^	1325 ppm	15%
M4: C0.02	221 ppm	Quiet	100% ^A^	1 ^B^	10% ^B^	891 ppm	25%

^A^ Compulsory use of face masks. ^B^ Obtained from local governmental data sources [73,74].

**Table 5:** Comparative performance of mobile robots embedding a CO_2_ sensor.

Characteristic	Jin [44]	Quintana [45]	Rahmaniar [46]	Yang [47]	Curea [48]	This Proposal
Autonomous	Yes	Yes	No	Yes	No/Yes	Yes
Human-sized	No	No	No	No	No	Yes
Omnidirectional	No	No	No	No	Yes	Yes
Tested with people	No	Yes	No	Yes	No	Yes
Multi-story navigation	No	No	No	No	No	Yes
Evaluated in COVID-19 pandemic	No	No	No	No	No	Yes

## Data Availability

The raw data supporting the conclusions of this article will be made available by the authors on request.
